# Peripheral injection of human umbilical cord blood stimulates neurogenesis in the aged rat brain

**DOI:** 10.1186/1471-2202-9-22

**Published:** 2008-02-14

**Authors:** Adam D Bachstetter, Mibel M Pabon, Michael J Cole, Charles E Hudson, Paul R Sanberg, Alison E Willing, Paula C Bickford, Carmelina Gemma

**Affiliations:** 1 Department of Molecular Pharmacology and Physiology, University of South Florida, College of Medicine, Tampa, FL 33612, USA; 2 Department of Neurosurgery, Center of Excellence for Aging and Brain Repair, University of South Florida, College of Medicine, Tampa, FL 33612, USA; 3 James A. Haley Veterans Administration Hospital, Tampa, FL 33612, USA; 4 Saneron CCEL Therapeutics Inc., Temple Terrace, FL 33617, USA

## Abstract

**Background:**

Neurogenesis continues to occur throughout life but dramatically decreases with increasing age. This decrease is mostly related to a decline in proliferative activity as a result of an impoverishment of the microenvironment of the aged brain, including a reduction in trophic factors and increased inflammation.

**Results:**

We determined that human umbilical cord blood mononuclear cells (UCBMC) given peripherally, by an intravenous injection, could rejuvenate the proliferative activity of the aged neural stem/progenitor cells. This increase in proliferation lasted for at least 15 days after the delivery of the UCBMC. Along with the increase in proliferation following UCBMC treatment, an increase in neurogenesis was also found in the aged animals. The increase in neurogenesis as a result of UCBMC treatment seemed to be due to a decrease in inflammation, as a decrease in the number of activated microglia was found and this decrease correlated with the increase in neurogenesis.

**Conclusion:**

The results demonstrate that a single intravenous injection of UCBMC in aged rats can significantly improve the microenvironment of the aged hippocampus and rejuvenate the aged neural stem/progenitor cells. Our results raise the possibility of a peripherally administered cell therapy as an effective approach to improve the microenvironment of the aged brain.

## Background

Aging is accompanied by a process of cellular senescence that occurs throughout the body, resulting in a decrease in the regenerative potential of the stem cell pools [1]. In the brain there are two stem cell pools, one residing in the subventricular zone (SVZ), and the other in the subgranular zone (SGZ) of the dentate gyrus of the hippocampus. As in other stem cell pools such as the hemapoietic pool in the bone marrow or the satellite stem cells in the muscle, the stem cells in the brain lose there capacity to generate new cells with age [2-4]. In the brain it appears that the decrease in neurogenesis is a result of a decrease in proliferation of the stem cells and not due to a loss of the cells [5]. In the muscle it has been shown that the stem cells can be rejuvenated by exposure of the cells to the systemic environment of a young animal through parabiosis [6]. Even though it has been known since the 1960s that a cellular senescence occurs with age [7], it is less clear if this cellular senescence leads to an aging phenotype, particularly to the age related cognitive decline.

However, it is clear that the process of cellular senescence that occurs with age is an important mechanism to protect against cancer. There are a number of tumor-supressor genes, including p53 and p16^ink4A^, which respond to cellular stressors to induce senescence [8]. It has recently been shown that knocking out p16^ink4A ^can restore the proliferative potential of the aged neural stem cells [9], but the animals have decreased longevity due to tumor formation [10]. This demonstrates the important balance that oncogenes play in protecting organisms from cancer, but with the negative consequence of inducing an aging state of cellular senescence. An effective target to lessen the amount of senescence might be the cellular stressors that accumulate with age which include telomere shorting [1], oxidative stress [11-13], inflammation [14], increased corticosteroid levels [15], and a decrease in a number of trophic factors including brain-derived neurotrophic factor (BDNF), vascular endothelial growth factor (VEGF), Insulin-like Growth Factor-1 (IGF-1) and fibroblast growth factor 2 (FGF-2) [16,17].

A potent cellular stressor that is increased with age is inflammation. Recently, our laboratory has shown that reducing neuroinflammation in aged rats by blocking the conversion of pro-IL-1β to IL-1β through inhibition of the converting enzyme caspase-1 rescued some of the age-related decrease in neurogenesis [18] and resulted in an improvement in cognitive function [19]. We believed that human umbilical cord blood mononuclear cells (UCBMC) may have a similar potential to restore some of the loss in capacity of the neural stem/progenitor cells ability to proliferate and differentiate into neurons.

In an animal model of stroke, UCBMC administered intravenously have reduced infarct volume and improved functional recovery on behavioral measures [20]. The effects of UCBMC have been attributed to changes in the microenvironment of the brain, through the release of trophic factors or by reducing inflammation, and not by a direct replacement of cells [21-23]. UCBMC contains a number of cell types including B-Cells and T-Cells, as well as, mesenchymal and endothelial progenitor cells. UCBMC is also a rich source of CD34^+ ^hematopoietic stem cells [24-26]. It was recently demonstrated that a systemic injection of UCBMC cells could suppress inflammation in the brain following stroke. Moreover, the effects of UCBMC cells seemed to shift the cytokine expression from a Th1 response to a Th2 response [20,23,27]. In addition to the immune modulatory effects, UCBMC cells also produce a number of trophic factors including, but not limited to, VEGF, nerve growth factor, and cytokine colony stimulating factor-1, thrombopoietin, and IL11 [20,28,29].

The goal of the present study was to determine if UCBMC could stimulate the endogenous stem/progenitor cells to regenerate new cells. To this end, young and aged rats were intravenously administered a single dose of UCBMC to determine if UCBMC could increase proliferation of the neural stem/progenitor cells as well as to determine if there would be an effect on neurogenesis in the aged rats. This study provides insight into how the aged stem cell niche could be rejuvenated. Furthermore, as the UCBMC are administered minimally invasively this study raises the possibility of a clinically applicable therapeutic for the aged brain.

## Results

### Human umbilical cord blood mononuclear cells (UCBMC) stimulate proliferation of the senescent hippocampal neural stem cell

**Figure 1:**
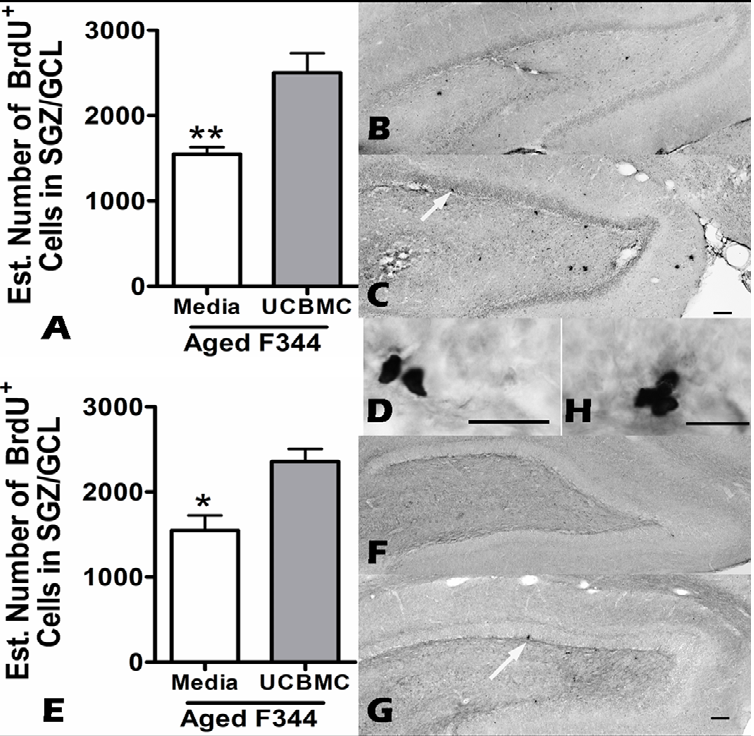
**Proliferation is increased in aged rats following UCBMC treatment**. To determine if UCBMC could stimulate proliferation of the hippocampal neural progenitor/stem cells rats received two i.p. injections of BrdU (50 mg/kg) and were sacrifice the following day. (A) Quantification of the BrdU immunoreactive cell in the SGZ/GCL in aged rats 2 days after the UCBMC treatment showed that there was a significant (p < 0.005) increase in the number of BrdU immunoreactive cells. (B, C) Photomicrographs of the dentate gyrus of a media-treated rat (B) and a UCBMC-treated rat (C) shows the BrdU staining in those animals sacrificed 2 days after the treatment. (D)The arrow in C points to a cluster of BrdU immunoreactive cells from the UCBMC-treated rat shown in D at higher magnification. (E) To determine how long proliferation might remain elevated injections of BrdU (50 mg/kg) began 14 days after the treatment. Quantification of the BrdU immunoreactive cells determine that the UCBMC-treated group had significantly (p < 0.01) more cells in the SGZ/GCL then the animals that received media alone. (F, G) BrdU staining of the media-treated (F) and the UCBMC-treated (G) animals in the dentate gyrus of the hippocampus 15 days after the treatment. (H) Arrow in G points to cells shown at higher magnification in H. (scale bar for B, C, F, G is 100 μm; scale bar for D, H is 25 μm)

In the first of a series of experiments we wanted to determine if UCBMC given intravenously could stimulate the proliferation of the endogenous stem/progenitor cells in the hippocampus. We chose to study proliferation as a slowing of the cell cycle and a decrease in proliferation seems to be most affected with age when compared to the ability of the cells to survive and differentiate into neurons which appears to occur at relatively the same rate in young animals [30]. The effect of a single intravenous injection of UCBMC on cell proliferation in the granule cell layer in young (3-months old) or aged (20-months old) F344 rats was determined by analyzing BrdU staining 24 hours after the BrdU injections (48 hours following UCBMC injection). Using the optical fractionator method of design based stereology [31], we found that in the aged animals there was a significant increase (t(9) = 4.256; p < 0.005) in the number of BrdU^+ ^cells in the UCBMC group (2504 ± 227.3 n = 5) compared to the animals that received media alone (1549 ± 82.07 n = 6) (Figure 1A). In young animals there was no significant effect of the UCBMC treatment (data not shown). To determine if there might be a prolonged effect on proliferation in the aged F344 rats BrdU injections were given 14 days after the UCBMC treatment. Figure (1E) shows the effect of a single intravenous injection of UCBMC on the number of cells that incorporated BrdU on day 14. Stereological analysis revealed that in the aged UCBMC-treated rats there was a significant increase in the number of BrdU^+ ^cells (t(12) = 3.468; p < 0.01) (2357 ± 149.4 n = 6) compared to the media-treated group (1548 ± 176.9 n = 4).

### Neurogenesis is stimulated in the aged hippocampus following UCBMC treatment

**Figure 2:**
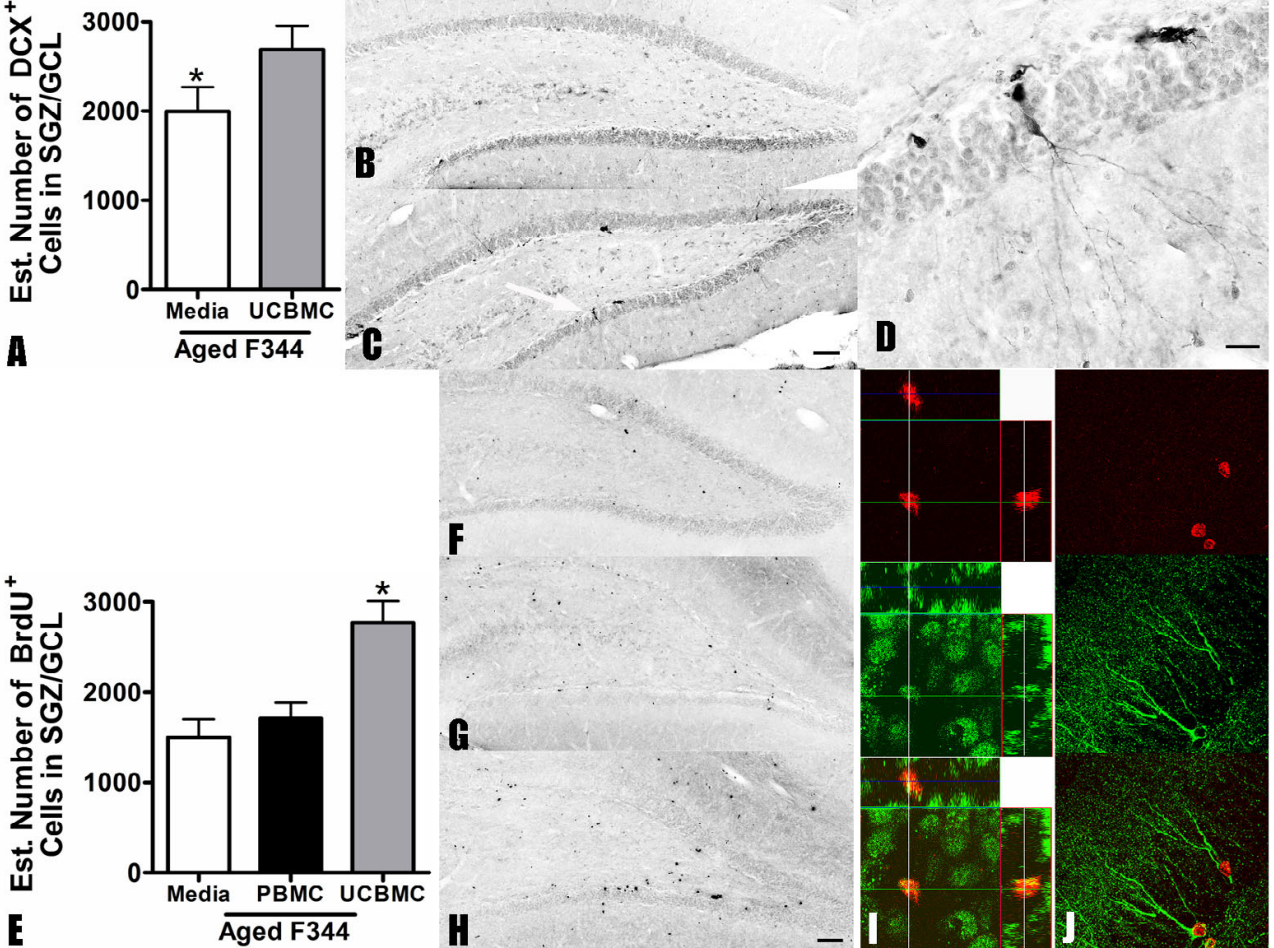
**15 days after a UCBMC treatment neurogenesis is increase in aged rats**. To determine if UCBMC treatment could stimulate neurogenesis aged F344 rats were sacrificed and immunohistochemical stained for DCX and BrdU. (A) A significant increase (p < 0.05) in the number of DCX^+ ^cells, quantified in the SGZ/GCL, was found in the UCBMC treated rats. (B, C) Photomicrographs show the dentate gyrus demonstrating the DCX immunohistochemistry in the media-treated (B) and UCBMC-treated (C) rats. (D) A higher magnification photomicrograph of area indicated in C shows a number of DCX^+ ^cells showing the different morphologies of the cells. (E) The results obtained with DCX were confirmed by BrdU. BrdU was injected i.p. for five consecutive days after the single injection of UCBMC. 10 days after the last injection of BrdU the animals were sacrificed. Compare to both a media control as well as an human adult peripheral blood (PBMC) control the UCBMC treated animals had significantly more BrdU^+ ^cells (p < 0.01). (F, G, H) Photomicorgaphs of dentate gyrus shows BrdU immunohistochemistry in the media-treated (F), PBMC-treated (G) and UCBMC-treated (H) rats. (I, J) Immunofluorescence was conducted to determine the phenotype of the BrdU^+ ^cells. (I) An example of the cells double labeled with BrdU^+^/NeuN^+ ^(I; shown in orthogonal projection) and BrdU^+^/TUJ1^+ ^(J; shown using maximum projection). (scale bar for B, C, F, G, H is 100 μm; scale bar for D is 25 μm)

To determine if UCBMC would also stimulate neurogenesis in the aged rats, doublecortin (DCX) immunostaining was examined. Counting the number of DCX^+ ^cells in the SGZ/GCL, we found a significant increase (t(16) = 2.188; p < 0.05) in the number of DCX^+ ^cells in the aged rats 15 days after a single i.v. injection of the UCBMC (2619 ± 212.6 n = 9) compared to animals that received media alone (1843 ± 283.9 n = 9) (Figure 2A). To confirm the results obtained by DCX, and to determine if there was any change in the ability of the proliferating cells to differentiate following a UCBMC treatment, we injected the animals with BrdU (50 mg/kg) for five days beginning 24 hours after our i.v. treatment. Quantifying the number of BrdU^+ ^cells in the SGZ/GCL using the optical fractionator method of design based stereology we found a similar increase in the number of BrdU^+ ^cells in the aged UCBMC treated group as was found using the neurogenic marker DCX (Figure 2A–D). In aged rats, there was a significant increase (F(2, 14) = 10.94, p < 0.005) in the number of BrdU^+ ^cells generated over a period of five days following a single i.v. injection of UCBMC (2772 ± 263.3 n = 5) compared to the rats that received media alone (1498 ± 206.1 n = 5); as determined by the Tukey's Multiple Comparison Test (p < 0.01). In this experiment, we also included a group that was injected with adult human peripheral blood mononuclear cells (PBMC) as a control for the effect of delivering cells. The PBMC group was determined to have significantly fewer BrdU^+ ^cells (1712 ± 171.2 n = 5) than the UCBMC treated group (p < 0.01), but this was not significantly different from the group that received media alone (Figure 2E–H). As with the results of the proliferation study, young rats showed no significant effect of UCBMC treatment (data not shown). To determine if the treatment with UCBMC might alter the phenotype of the newborn cells, we double labeled with the antibodies to Tuj1, NeuN and GFAP. While exhaustive sampling was not conducted, 50 BrdU^+ ^cells were analyzed from each rat (4 rats per group) using confocal microscopy for each marker and there did not appear to be any change in phenotype due to the treatment (Figure 2I–J). To confirm that the increase in neurogenesis was from the endogenous stem/progenitor cells, sections were stained for HuNu to look for the presence of the transplanted cells in the brain. Cells positive for the HuNu were found in the blood smears of the rats that were treated with UCBMC, but no HuNu immunoreactive cells were found in the hippocampus of the rats (data not shown).

### A decrease in microglia activation following UCBMC correlates with the increase in neurogenesis

**Figure 3:**
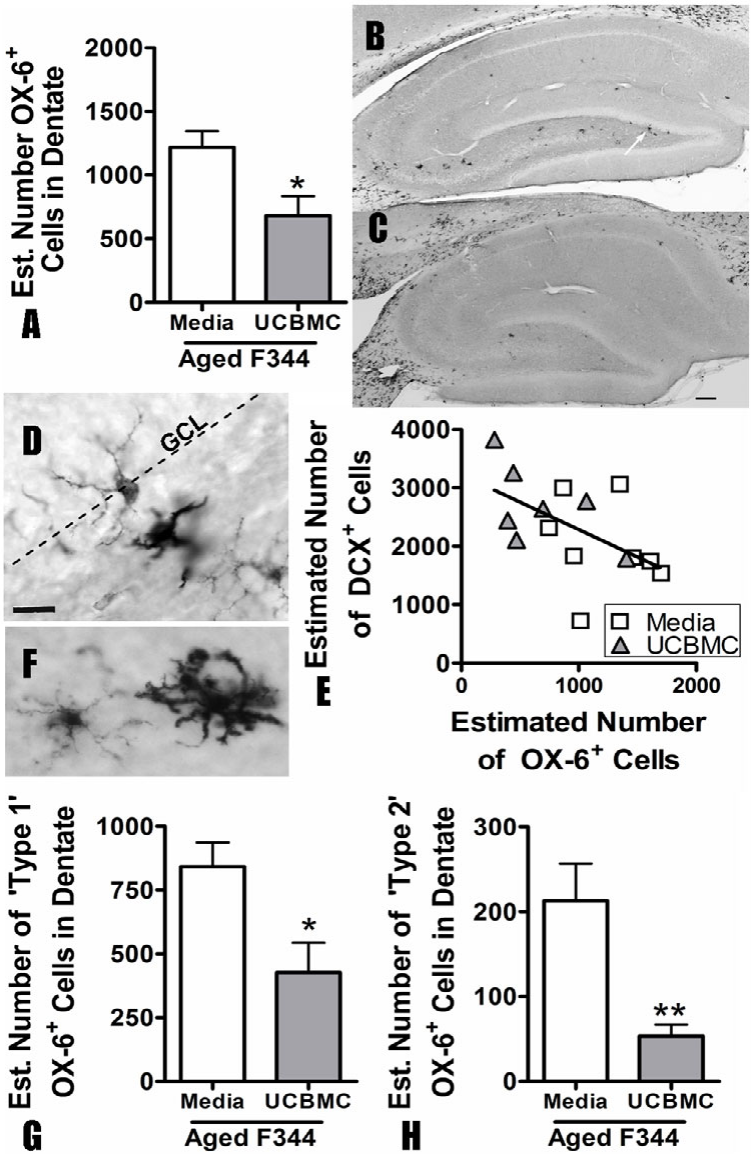
**The decrease in microglia activation correlates with neurogenesis**. 15 days after the UCBMC treatment a significant reduction (p < 0.05) was found in the number of OX-6^+ ^cells in the dentate gyrus of the aged rats (A). (B, C) Photomicrographs are shown of the hippocampus of media-treated (C) and UCBMC-treated (C) rats. (D) A higher magnification photomicrograph of area indicated by arrow in B. (E) A significant negative correlation (p < 0.01) was found between the number of OX-6^+ ^cells and the amount of neurogenesis as determine by the number of DCX^+ ^cells. (F) The OX-6^+ ^were further characterized based on morphology. The cell on the left represents a typical 'Type 1' cell the cell on the right represents a typical 'Type 2' cell. Both 'Type 1' (p < 0.05; G) and 'Type 2' (p < 0.01; H) OX-6^+ ^cells were significantly reduced in the aged animals following UCBMC treatment, but there was a greater reduction in 'Type 2' cells amounting to a four fold change. (scale bar for B, C is 200 μm; scale bar for D is 25 μm)

Using the optical fractionator method of design based stereology, we counted the number of OX-6^+ ^cells in the dentate gyrus 15 days after a single UCBMC injection; this was at the same time point that we observed an increase in DCX^+ ^cells and BrdU^+ ^cells. OX-6 is a marker for MHCII and presumably stains for microglia in an activated, proinflammatory state. In aged rats, we found that 15 days after the UCBMC treatment there was a significant decrease (t(12) = 2.699; p < 0.05) in the total number of activated OX-6^+ ^microglia in the UCBMC group (678.7 ± 155.3 n = 7) compared to the media control (1217 ± 128.0 n = 8) (Figure 3A). The decrease in OX-6^+ ^microglia negatively correlated with the number of DCX^+ ^cells (Spearman r(15) = ^-- ^0.6429; p < 0.01) (Figure 3E).

Morphologically the OX-6^+ ^cells expressed two main phenotypes (see Figure 3F). Type 1 microglia appear to be in a more quiescent state based on morphology Type 2 microglia were thought to represent a more activated state. The type 1 microglia make up the majority of the OX-6^+ ^cells in the dentate gyrus and were found to be significantly decreased (unpaired t(12) = 2.791; p < 0.05) in aged rats following UCBMC treatment (426.6 ± 117.0 n = 7) compared to controls (842.4 ± 94.67 n = 8) (Figure 3G). The type 2 microglia, while representing a smaller percentage of the total OX-6^+ ^microglia, were significantly reduced (t(12) = 3.281; p < 0.01) to a greater extent by the UCBMC treatment (53.30 ± 13.27 n = 7) compared to controls (212.9 ± 43.82 n = 8) than the total microglia (Figure 3H). Fifteen days after the UCBMC treatment, there was a 4 fold change in the number of type 2 OX-6^+ ^microglia, whereas there was only a 1.8 fold change in the total number of OX-6^+ ^microglia. It appears that the highly activated microglia are being reduced to a greater extent by the UCBMC treatment, although all OX-6^+ ^microglia are affected.

## Discussion

The present study explored whether human umbilical cord blood mononuclear cells (UCBMC) could improve the neurogenic niche of the aged brain and stimulate the endogenous stem/progenitor cells to generate new neurons. As determined by stereological analysis of both DCX and BrdU, a single peripherally administered injection of UCBMC appeared to stimulate neurogenesis. The finding that the administration of UCBMC also increased the number of proliferative cells generated within 24 hours following the treatment, suggests that the increase in neurogenesis observed in this study may be a consequence of an increase in proliferation rather than changes in differentiation or survival of newly generated cells. To support this hypothesis, it will be important to allow more time for the cells to fully mature and then determine if there is still no change in the phenotype of the BrdU^+ ^cells. It will also be important to determine what effect UCBMC have on the survival of the BrdU^+ ^cells.

In addition, it was determined that UCBMC were able to increase cell proliferation for at least fifteen days in the aged rats. This suggests that the UCBMC may have a beneficial effect on the microenvironment of the aged brain. In support of this hypothesis we show that coinciding with an increase in neurogenesis in the aged treated rats, there was a decrease in the number of activated microglia in the dentate gyrus. A negative correlation between the degree of inflammation as indicated by the activation of microglia and the number of newborn neurons has been previously described [32]. Consistent with previous studies showing that UCBMC have the potential to reduce neuroinflammation [20,23,27,30] in the aged brain, we did find that neurogenesis correlated with the number of activated microglia, suggesting that UCBMC were stimulating neurogenesis by decreasing microglia activation. Although other possibilities cannot be excluded, since UCBMC could be having multiple effects including increasing trophic support as previously published [20,28,29].

UCBMC have been shown to reduce neuroinflammation [20,23,27,30] and, consistent with previous studies, we show here that the peripherally administered UCBMC do have anti-inflammatory properties. It appears that one of the factors that leads to the negative regulation of neural stem cells is inflammation [32-34]. A primary source of inflammation in the CNS is from the macrophages/microglia which can produce a wide array of cytotoxic factors, including proinflammatory cytokines such as tumor necrosis factor (TNF), IL-1, IL-6 and IL-12 [35,36]. With age, microglia shift from a quiescent state into an active proinflammatory state. It is not clear if this change in activation state is in response to injury, infection, or debris or if it is due to dysregulated cytokine levels. Another possibility recently proposed, is that microglia becoming senescent and this leads to them becoming dysfunctional [37,38]. It has previously been demonstrated in models of induced inflammation through the use of LPS or radiation, a dramatic decrease in proliferation and neurogenesis occur, and when the inflammation is alleviated the replicative potential of the stem cells returns [32,34]. This effect is likely a protective mechanism so that the DNA is not exposed to the noxious inflammatory environment which could damage the replicating DNA. This correlation also imparts support to the hypothesis that UCBMC stimulate neurogenesis by decreasing inflammation, particularly the activation state of microglia. However, it does not rule out the possibility that UCBMC may be acting on multiple targets, with microglia only representing one part of the total mechanism.

While UCBMC do seem to have an effect on microglia, it is not clear how this occurs. A number of studies have shown that T-cells appear to act on macrophages/microglia to cause them to adopt a phenotype that is 'pro-repair' (i.e. the macrophages/microglia: clear debris, buffer toxic compounds, and produce growth factors), without being pro-inflammatory (i.e. producing TNF-α, NO, or COX-2) and this effect can promote neurogenesis and be neuroprotective [39-43]. As T-cells are a major fraction of UCBMC, it is possible that the naïve T-cells in the UCBMC are able to induce a protective T-cell mediated response in the aged rats, since adult PBMC did not have an effect. Alternatively, the CD34^+ ^stem cells in the UCBMC may be involved. Taguchi et al. [29] has shown that CD34^+ ^stem cells can increase both angiogenesis and neurogenesis as part of the protective mechanism against stroke. From the results of the current study it can not be determined if the effects of the UCBMC are a result of direct action on the brain or though peripheral effect. However, the fact that we did not detect any immunoreactivity for human nuclei in the brains of the UCBMC-treated rats raises the possibility that the UCBMC may be acting through a peripheral mechanism. Moreover, the observation that the adult PBMC did not alter hippocampal neurogenesis ruled out the possibility of a non-specific effect due to an influx of cells, supporting our belief that the increase in neurogenesis, which occurred following treatment with UCBMC was not due to an influx of cells but was specific to UCBMC.

The present study did not attempt to determine if decreasing senescence of the neural stem cells could reverse the cognitive decline with age. There is still much debate surrounding the role of neurogenesis in learning and memory [44-49] and whether cellular senescence of the stem cell pool with age leads to an aging phenotype. While not a goal of the current study, it will be important to determine if the rejuvenation of the aged stem/progenitor cell pool can reverse the age-related cognitive decline.

In summary, this study demonstrates that a single peripheral injection of UCBMC could stimulate the endogenous neural stem/progenitor cells to increase proliferation. We also determined that the UCBMC were able to improve the microenvironment of the aged brain by reducing the number of activated microglia, and this reduction is correlated with an increase in neurogenesis. Further work will be important to determine the mechanism of action of UCBMC in the aged rats, including the possible role of the immune system in a T-cell mediated response, as well as the affects of angiogenesis via the CD34^+ ^stem cells. It will also be important in future experiments to determine the duration that a single injection of UCBMC will elevated proliferation in aged rats. Not only do the results of this study provide novel insight into the state of the aged stem cell niche, the ability of the UCBMC to exert their effects while being administered minimally invasively may make translation to the clinical setting more likely. For this reason it will be important in future studies to determine the most efficacious dose and dosing regimen. Nevertheless, this is the first time that a systemic injection of hematopoietic cells has been shown to restore the regenerative potential of the aged brain, providing a novel insight into how the regenerative potential of the aged stem cell niches could be restored.

## Conclusion

The results demonstrate that a single intravenous injection of UCBMC in aged rats can significantly improve the microenvironment of the aged hippocampus and rejuvenate the aged neural stem/progenitor cells. Our results raise the possibility of a peripherally administered cell therapy as an effective approach to improve the microenvironment of the aged brain.

## Methods

### Cell preparation

Cryopreserved human umbilical cord blood mononuclear cells (UCBMCs) were obtained from Saneron CCEL Therapeutics, Inc. (Tampa, FL, USA). Cryopreserved Human peripheral blood cells (PBMC) (mononuclear fraction) were obtained from AllCells, LLC (Emeryville, CA, USA). Just prior to intravenous (i.v.) injection, the UCBMC or PBMC were thawed into media (Hanks' balanced salt solution, HBSS, Gibco) at 37°C, washed, and the number of viable cells was determined using the trypan blue exclusion method [23]. Cell viability ranged from 85 to 88%. Cell concentration was adjusted to 10^6 ^viable cells/500 μl. Rats were then anesthetized with 3% isofluorane and randomly chosen to receive a single i.v. injection via the penile vein of UCBMC at a dose 10^6 ^cells shown most effective in a stroke model [22], 10^6 ^PBMC, or media for both the aged and young rats.

### Animals

All experiments were conducted in accordance with the National Institute of Health Guide and Use of Laboratory Animals, and were approved by the Institutional Animal Care and Use committee of the University of South Florida, College of Medicine. Male Fisher 344 (F344) rats (NIA contract colony, Harlan Sprague Dawley, Indianapolis, IN), were pair-housed in environmentally controlled conditions (12:12 h light:dark cycle at 21 ± 1°C) and provided food and water *ad lib*. Two age groups of animals young (3 months old) and aged (20 months old) were used in this study. The mean life span of the F344 rats is approximately 29 months with a maximal life span of 36 months [50].

Rats were then divided in three groups. Group 1 received 50 mg/kg of bromodeoxyuridine (5-bromo-2-deoxyuridine, BrdU; Sigma, St. Louis, MO, USA), intraperitoneal (i.p.) twice a day beginning 24 hours the injection of UCBMC, and were sacrificed the subsequent day. Rats in group 2 received BrdU (50 mg/kg, i.p.) twice a day, beginning fourteen days after the administration of UCBMC and were sacrificed on the following day. Rats in Group 3 received BrdU (50 mg/kg, i.p.) for five consecutive days, beginning the day after the administration of UCBMC and were the sacrificed day fifteen.

### Tissue collection and processing

The rats were anesthetized with pentobarbital (50 mg/kg, i.p.). Blood was collected by cardiac puncture and smears were made of the blood to look for the presence of the transplanted cells. The rats were transcardiac perfusion with phosphate-buffered (PB), followed by 4% paraformaldehyde in PB. The brains were postfixed in 4% paraformaldehyde for 12 h, after which they were transferred into 30% sucrose in phosphate-buffered saline (PBS) for at least 16 h, and stored at 4°C. Exhaustive sagittal sections of the left hemisphere were made, at 40 μm using a Microm cryostat (Richard-Allan Scientific, Kalamazoo Michigan) and stored in cryoprotectant at 4°C.

### BrdU Immunohistochemistry

All immunohistochemical staining was conducted on free-floating sections for every sixth section for the entire hippocampus beginning with a random start and including sections before and after the hippocampus to ensure that the entire structure was sampled; with one exception, in the aged animals from group 3 a one in three series was stained to allow for sampling of an adequate number of BrdU^+ ^cells. For BrdU staining, sections were pretreated with 50% formamide/2× SSC (0.3 M NaCl, 0.03 M sodium citrate) at 65°C for 2 hours, rinsed in 2× SSC, incubated in 2 N HCL for 30 minutes at 37°C, washed with borate buffer (pH 8.5), then PBS. This was followed by quenching endogenous peroxidase activity in 0.3% H_2_O_2 _solution in 30% methanol; then one hour in blocking solution (0.1 M PBS supplemented with 3% normal horse serum and 0.25% Triton X-100: PBS-TS); followed by incubation overnight with mouse-anti-rat-BrdU (1:100; Roche) in PBS-TS. The following day the sections were washed and then incubated for one hour in a biotinylated secondary antibody (1:200; Vector Laboratories, Burlingame, CA) in PBS-TS; then washed before one hour incubation in avidin-biotin substrate (ABC kit, Vector Laboratories, Burlingame, CA); and then washed before 10 minutes incubation in DAB solution (Vector Laboratories, Burlingame, CA). Sections were then mounted onto glass slides and coverslipped with mounting medium.

### Doublecortin and OX-6 Immunohistochemistry

Doublecortin (DCX) is a marker of migrating neurons that is expressed for approximately three weeks after the cell is born and has been shown to be a reliable indicator of neurogenesis [51,52]. For DCX immunohistochemistry a polyclonal goat antibody raised against human DCX (1:200; SC-8066, Santa Cruz biotechnology, Santa Cruz, CA, USA) was used following a similar protocol to BrdU except the antigen retrieval steps were omitted and Goat serum (Vector Laboratories, Burlingame, CA) was used instead of horse serum. For OX-6 immunohistochemistry a monoclonal antibody directed against the rat major histocompatibility II (MHCII) (RT1B, Becton, Dickinson Pharmingen, San Diego, CA, USA) was used at a concentration of 1:750 in place of the other primary antibodies all other steps were the same.

### Immunofluorescence

Tissues were pretreated with 2 N HCL for 2 hours at room temperature, washed, and incubated in blocking solution (0.1 M PBS containing 10% goat serum and 0.3% Triton X-100) for 1 hour at room temperature. Tissues were then incubated in rat anti-BrdU (1:400; Accurate Chemical, Westbury, NY) and additional primary antibodies [anti-GFAP (1:500; Dako, Carpinteria, CA), mouse anti-NeuN (1:100; Chemicon, Temecula, CA), mouse anti-TUJ1 (1:800; Convance, Berkeley, CA)], overnight at 4°C. Tissues were then rinsed 3 times in PBS and the appropriate secondary antibody conjugated to an Alexafluor probe (Molecular Probes, Eugene, OR) was applied for 2 hour. Following 6 washes in PBS, tissues were mounted on slides and coverslipped using Vectashield (Vector Labs, Burlingame, CA).

### Human Nuclei immunofluorescence

To detect for the presence of the transplanted cells, blood smears and tissue sections were stained with a mouse monoclonal antibody that recognizes Human Nuclei antigen (HuNu) (MAB 1281; 1:50; Chemicon, Temecula, CA), and does not react with rat nuclei. Prior to incubation overnight at 4°C in the HuNu antibody, the samples were washed in PBS and incubated in blocking solution (0.1 M PBS containing 10% goat serum and 0.3% Triton X-100) for 1 hour at room temperature. The HuNu antibody was visualized by secondary antibody conjugated to an Alexafluor probe (Molecular Probes, Eugene, OR).

### Quantification and imaging

To determine cell numbers the optical fractionator method of unbiased stereological cell counting techniques [31] was used with a Nikon Eclipse 600 microscope and quantified using Stereo Investigator software (MicroBrightField, Colchester, VT). For the proliferation study, because of the low number of BrdU^+ ^cell in the aged animals the virtual grid and counting frame were both 125 μm × 125 μm in order to count all the cells that were present in the section. For all other counts sampling was optimized to count at least 200 cells per animal with error coefficients less than 0.07. Outlines of the anatomical structures were done using a 10×/0.45 objective and cell quantification was conducted using a 60×/1.40 objective. OX-6^+ ^cells were counted in the entire dentate gyrus including the subgranular zone (SGZ: defined as a two cell diameter band on both sides of the granular cell layer (GCL)). All other cell counts were done in the SGZ/GCL. The phenotype of the BrdU^+ ^cells were analyzed using an inverted Zeiss LSM 510 confocal microscope with a 40×/1.30 NA oil immersion objective. Argon and HeNe laser lines in conjunction with 488 and 555 band pass filters were applied to excite the samples using line switching to minimize crosstalk between fluorochromes. Images and Z-stacks were produced with dual photomultiplier detectors and the LSM 5 version 3,2,0,115 software suite, and optical Z stacks where created at 2 μm intervals throughout the 40 μm of the sections with a guard region of 2 μm excluded from top and bottom of the Z stack. The Z stacks were rotated in all planes to verify double labeling.

### Statistical analyses

Data are presented as mean cell number ± SEM. Statistical analysis was performed using an unpaired, two-side *t*-test, or a one-way ANOVA followed by a Tukeys *post-hoc *test. *p *< 0.05 was considered to be significant.

## Abbreviations

GCL (granular cell layer), PBMC (peripheral blood mononuclear cells), UCBMC (umbilical cord blood mononuclear cells), SGZ (subgranular zone)

## Competing interests

PCB, AEW are consultants to Saneron CCEL Therapeutics Inc (SCTI). PRS is a co-founder of SCTI. AEW & PRS are inventors of UCBMC related patents applications.

## Authors' contributions

ADB, PRS, AEW, PCB, CG designed research. ADB, MMP, MJC, CEH, CG performed research. ADB wrote paper. All authors read and approved the final manuscript.
